# Nutrition, Sleep, Circadian Rhythms, and Health Implications: “Come Together”

**DOI:** 10.3390/nu14235105

**Published:** 2022-12-01

**Authors:** Egeria Scoditti, Sergio Garbarino

**Affiliations:** 1 National Research Council (CNR), Institute of Clinical Physiology (IFC), 73100 Lecce, Italy; 2 Department of Neuroscience, Rehabilitation, Ophthalmology, Genetics and Maternal/Child Sciences (DINOGMI), University of Genoa, 16132 Genoa, Italy

Over the last few years, novel and important aspects of nutrition that are often overlooked in nutritional epidemiology, experimental research, and recommendations for health maintenance and disease prevention concerning the circadian rhythmicity of feeding, as well as the bidirectional interaction of nutrition with central and peripheral endogenous circadian clocks, and with other rhythmic behaviors including the sleep–wake cycle have received increasing attention from the research community. Besides total energy intake and diet composition, the daily rhythms of eating patterns are an integral part of homeostasis [1]. Feeding is a resetting and entraining cue for the circadian clock [2]. Unbalanced nutrition and irregular meal timing patterns, which occur in today’s modern 24/7 society, especially due to night shifts or social jet lag, are risk factors for circadian disruptions in physiological processes, including metabolism, neurologic, immune, cardiovascular, and endocrine functions, among others, thus enhancing the risk for numerous chronic diseases [1]. Furthermore, dietary intake and timing have a significant impact on sleep quality and duration and can therefore increase the risk of sleep disturbance and related health outcomes [3]. Conversely, sleep patterns influence eating behavior, diet quality, and total energy intake [4].

The importance of these aspects become more evident when considering the significant implications for public health, disease prevention, and disease management that arise from the altered crosstalk between diet, sleep, and the circadian system due to genetics, unhealthy behaviors, (patho)physiological states, and/or environmental factors. Notwithstanding this, the integrated role of diet, sleep, and circadian rhythms is not accounted for in public health policy or in health promotion plans, thus representing a current gap that should be filled.

This Special Issue, entitled “Nutrition at the Interface of Sleep and Circadian Rhythms: Implications for Health” focused on the interaction as well as the related health implications of nutrition with sleep and circadian rhythms, gathering 12 papers, including eight original research articles and four reviews.

The prospective study performed by Al-Musharaf et al. [5] investigated sleep patterns among a cohort of 140 Saudi women aged 18–39 years old throughout all three trimesters of pregnancy. They found worsening sleep quality and a shortened sleep duration from early to late pregnancy, with low socio-economic status, low serum vitamin D levels, greater energy intake, and sitting time as significant and independent predictors of worsening changes in sleep patterns during pregnancy.

The study by Fenton et al. [6] was a three-armed randomized controlled trial in 116 adults (70% female, 44.5 years) with overweight and obesity exploring the efficacy of a multi-component m-Health weight-loss intervention (enhanced) targeting sleep, diet, and physical activity to improve dietary intake over 6 months, and after a 12-month follow-up, compared to a waitlisted control group and the traditional dietary and physical activity intervention, the multi-component weight-loss intervention resulted in reduced total energy and sodium intake as well as increased fruit intake in adults at six months compared to the control group. Importantly, the enhanced intervention group improved their dietary intake relative to the traditional group at 12 months, with a higher intake of nutrient-dense foods and protein and a lower intake of energy-dense nutrient-poor foods.

Another prospective study conducted in 607 individuals aged 60 years of age or older found that a higher energy intake at dinner compared to during other eating occasions was a risk factor for the development of metabolic syndrome, mostly due to abdominal obesity and hypertriglyceridemia [7]. This study confirms the impact of meal timing on cardiometabolic health.

The short-term longitudinal study by Mathew et al. [8] assessed the bidirectional relationship between caffeinated beverage consumption and objective and subjective sleep in adolescents. Adolescents with a more variable sleep duration and midpoint were found to have higher average odds of consuming caffeinated beverages on a given day. The consumption of one or more caffeinated beverages predicted later sleep onset that night and a later wake time the next morning versus no consumption. These data could be important when considering that adolescents commonly report poor sleep patterns and increasingly adopt the habit of consuming caffeine, which may have a significant influence on sleep health.

Another paper [9] pertains the effect of energy balance on sleep in adolescents. In that study, 28 male adolescents, 14 with obesity and 14 normal-weight age-matched controls, underwent an experimental protocol that included a controlled balanced diet adjusted to energy requirements (eucaloric) and a diet offered ad libitum for three days in random order, after which sleep was measured by polysomnography. The results indicate that the eucaloric diet was able to improve sleep features, including sleep latency and N1 stage, in adolescents with obesity compared to the ad libitum diet. Interestingly, sleep improvements occurred in the absence of any substantial modification in macronutrient proportions and were correlated to reduced energy intake, especially during the evening meal.

The study by Strojny et al. [10] pointed to the effect of chronotype, a measure of the interindividual variability in circadian rhythmicity as reflected in the preferred timing of the sleep–wake cycle, on the effectiveness of a 3-week weight loss intervention (caloric restriction) in 131 adults with obesity. At the end of the intervention, both the morning and evening chronotypes reported a similar body weight and body mass index (BMI) reduction, though the evening chronotype, who had earlier meal and sleep times than their usual habits imposed on them, experienced a tendency towards greater losses in body fat compared to the morning chronotype.

Another randomized cross-over trial in female adolescents with the habit of skipping breakfast tested the effects of breakfast consumption compared to breakfast omission for seven days on free-living physical activity energy expenditure and dietary intake [11]. Physical activity and energy expenditure during physical activity did not differ between breakfast consumption and breakfast omission, and the total daily energy intake was almost identical between the conditions. However, breakfast omission was associated with an increased perceived morning appetite and a tendency for increased carbohydrate intake as an energy-intake compensation behavior. Consuming breakfast, on the contrary, contributed to higher fiber intake in breakfast-skipping adolescent girls.

Pacifico et al. [12] analyzed the effects of dietary patterns naturally rich in photosensitizers, such as vegan and vegetarian diets, on skin sensitivity to narrow-band ultraviolet B (NB-UVB) phototherapy in 119 adults suffering from psoriasis, a chronic inflammatory skin disease that is also characterized by circadian rhythmicity disorders. They found that vegan and vegetarian diets and, in particular, the intake of furocumarins are associated with greater skin sensitivity to NB-UVB phototherapy in psoriatic patients compared to omnivores, thus limiting the total number of phototherapy sessions needed. The study points to an important diet-related effect on psoriasis treatment, with implications for patient management and clinical outcomes.

Accordingly, among the published reviews, Controne et al. [13] analyzed the bidirectional associations of psoriasis with diet as well as sleep. Scientific evidence was discussed regarding the effect of unhealthy diets, mainly the Western diet, and of sleep disorders on psoriasis risk, development, and clinical outcomes, with the analysis of potential underlying common mechanisms involving an alteration of immune-mediated responses and chronic inflammation. The review draws attention to the importance of patient lifestyles and sleep patterns in the prevention and/or treatment of psoriasis.

In the review by Sejbuk et al. [14], modifiable factors affecting sleep quality were discussed, addressing, in particular, the role of nutrition, stimulants, and physical activity. The gathered data suggest that proper nutrition, in terms of caloric intake, micro- and macronutrient composition and balance as well as eating timing, may benefit sleep quality, while stimulants, including alcohol, nicotine, caffeine, and cannabis, negatively influence sleep quality. Physical activity and, in particular, a sufficient amount of moderate- to high-intensity exercise, preferably not performed in the late evening, emerges as an important player in improving sleep quality and preventing insomnia. Pending future research addressing individual lifestyle factors could be a strategy to significantly modulate sleep and, through this, to impact wellbeing and disease prevention.

In line with this notion, Scoditti et al. [15] synthetized evidence from the literature on the effect of the Mediterranean diet, one of the most studied and healthful dietary patterns, on sleep features in healthy individuals. Evidence, though limited to epidemiological studies, converge upon a benefit of the Mediterranean diet on sleep, promoting adequate sleep duration and quality and preventing sleep disturbances. The plausible mediating role of the antioxidant, anti-inflammatory, immunomodulatory, and neuroprotective properties of the foods and nutrients in the Mediterranean diet as well as the modulation of the gut microbiota in sleep improvement was described. The evidence further strengthens the notion that diet may influence sleep and could contribute to sleep hygiene.

Garbarino et al. [16] reviewed evidence from the literature regarding the role of nutrition (composition and timing) as a (de)synchronizer of the internal circadian clock in peripheral tissues, with the possible role of chrononutrition in contrasting the effects of circadian misalignment and sleep deprivation, which prevails in modern society, mainly due to shift work schedules and social jet lag. As an excellent example, chocolate, an energy-dense nutrient-rich food containing high levels of flavonoids, was discussed regarding its beneficial effect on mental and cognitive functions, the cardiovascular system, and metabolism as well as its attenuation of the stress- and sleep-deprivation-induced alteration of the circadian sleep–wake rhythm, especially when consumed at breakfast and generally during the active phase.

This Special Issue provides novel knowledge on the complex interplay of diet, sleep, and circadian rhythms and contributes to spurring further research as well as elaborate novel directions for improving public and individual wellbeing and health and strategies for the prevention of major diseases.

## Data Availability

Not applicable.
